# Kinds of Replication: Examining the Meanings of “Conceptual Replication” and “Direct Replication”

**DOI:** 10.1177/17456916211041116

**Published:** 2022-03-04

**Authors:** Maarten Derksen, Jill Morawski

**Affiliations:** 1 Department of Theory & History of Psychology, Faculty of Behavioural and Social Sciences, University of Groningen; 2 Psychology Department, Wesleyan University

**Keywords:** replication, crisis, performativity, multiplicity, epistemology

## Abstract

Although psychology’s recent crisis has been attributed to various scientific practices, it has come to be called a “replication crisis,” prompting extensive appraisals of this putatively crucial scientific practice. These have yielded disagreements over what kind of replication is to be preferred and what phenomena are being explored, yet the proposals are all grounded in a conventional philosophy of science. This article proposes another avenue that invites moving beyond a discovery metaphor of science to rethink research as enabling realities and to consider how empirical findings enact or perform a reality. An enactment perspective appreciates multiple, dynamic realities and science as producing different entities, enactments that ever encounter differences, uncertainties, and precariousness. The axioms of an enactment perspective are described and employed to more fully understand the two kinds of replication that predominate in the crisis disputes. Although the enactment perspective described here is a relatively recent development in philosophy of science and science studies, some of its core axioms are not new to psychology, and the article concludes by revisiting psychologists’ previous calls to apprehend the dynamism of psychological reality to appreciate how scientific practices actively and unavoidably participate in performativity of reality.

Since 2011, psychology has been experiencing a period of turmoil that is often referred to as a “crisis.” Methodology, statistics, theory, publication practices, and incentive structures have all become topics of often heated debate. Replication in particular is cast as a central issue, one shared by other sciences. It is noteworthy that the current troubles are referred to as both a “crisis of confidence” (e.g., Pashler & Wagenmakers, 2012) and as a “replication crisis” (e.g., Pashler & Harris, 2012). Failed replications have been a major factor in denting trust in the solidity of the discipline’s accumulated findings, which have been referred to as “a vast graveyard of undead theories” (Ferguson & Heene, 2012). What has been reported in psychology journals is thought to consist to a significant extent of false positives: the product of sloppy methods (e.g., low-powered studies) in combination with selective reporting and publication bias and/or other questionable research practices, such as hypothesizing after the results are known (i.e., HARKing; Kerr, 1998), and in some cases outright fraud. Although most subfields of psychology have been subject of these reports, some, like social psychology, are receiving greater attention.

But replication also figures prominently in the solutions to the problems that have been proposed: Many researchers hold that only when replication is made a standard element of the research process can confidence be restored and will psychology live up to its status as a science. Objectivity entails reproducibility, and testing the reproducibility of an effect in a replication study is a crucial part of science, an idea that is often attributed to Karl Popper (e.g., Srivastava, 2014a). What psychology needs, therefore, is more attention to replication and specifically to what is usually termed “direct replication”: repeating the experimental procedure of the original experiment as closely as possible to test whether it produces the same result. Over the past 10 years, many psychologists have taken up this challenge, often collaborating in large-scale replication projects involving dozens of researchers (Nosek et al., 2021).

There are some, however, who are critical of the emphasis on direct replication in the current debate and believe that it is misguided to strive for the reproducibility of effects. These authors argue that replication is not as rare as it is made out to be by the alarmist critics, but that it usually takes the shape of so-called “conceptual” replications, in which the same hypothesis or theory is tested but in a different way.^1^ This practice is defended with two related arguments. First, it is pointed out that psychology is not about behavioral phenomena, per se, but about the psychological processes underlying them. It is these processes that psychology’s theories describe. Second, it is argued that behavior is sensitive to context, and this context is socially, culturally, and historically highly variable. We therefore cannot expect the same experimental manipulation to have the same effect in different circumstances. Because of this context sensitivity, failure of a direct replication is not informative. The proper way to bolster and extend a theory is by conceptual replication. Whereas proponents of direct replication present theory as constrained (by evidence), advocates of conceptual replication accord theory a more central place in research.

In this article, we offer a perspective on psychological research, replication in particular, in which research is understood primarily as the production of effects, phenomena, and events rather than as the discovery of underlying mechanisms. We invite readers to envision how the epistemic premises of this perspective depart from conventional philosophy of science but retain a commitment to realism. Research can fruitfully be regarded as performative, in the sense that it creates (multiple) realities rather than that it discovers (a single) reality. We argue that such a perspective suggests a way forward beyond the increasingly unhelpful dichotomy of direct and conceptual replication and the disputes over what constitutes the “good-enough” replication it engenders. It also brings into focus the political dimension of psychological research and its “real-world” applications, both relatively underrepresented issues in the current debate in psychology.

Although performativity and, specifically, enactment theory may seem an outlandish or even “postmodern” perspective to some, we will also note that there are similarities with ideas and proposals put forward earlier by reputable psychologists such as William McGuire, Anthony Greenwald, and Paul Rozin. Notwithstanding the diversity of their views, they shared an emphasis on multiplicity and on research as a process primarily of making things happen, of producing effects. Each warned against an exclusive focus on science as theory testing, instead favoring a result-centered approach. In the discussion we will explore these connections further.

## From Representing to Enacting

Science is conventionally understood as generating accurate representations of an ordered, singular world; thus, psychological science aims to provide accurate representations of ordered patterns of thought, feeling, and behavior. This commonly held epistemic premise that the world is singular, ordered, and relatively stable—that reality is “out there” and that it can be discovered and represented through science—has motivated substantial research in science studies (also referred to as science and technology studies [STS]). Over the past 4 decades, STS researchers have examined scientific practices, not simply its theories. In so doing, they departed from the conventional view of science as “above all, a body of representations of reality” and moved toward “an understanding of science as a mode of performative engagement with the world” (Pickering, 2010, p. 19). In other words, representation is relocated “from the theoretical to the practical side of science” and thus is “no longer regarded as a propositional account of the world but as the activity of instrumentally producing traces, images and artifacts, which enable scientists to better grasp and handle the objects of their investigations” (Langlitz, 2015, p. 20). This epistemic move marks an analytic shift from scientific theories (representations) to scientific practices, from science as contemplation of the world to science as activity, and from science as writing (as texts) to science as doing. By approaching science as practice, researchers have closely investigated the extensive and difficult scientific work that culminates in representations of objects, concepts, and facts (Latour & Woolgar, 1986; Lynch & Woolgar, 1990; Pickering, 1994).

The focus on scientific practices and the ways they actively engage with and intervene in the world has been extended to consider how scientific entities are coextensive with scientific practices. Accordingly, objects, processes, or entities are understood as enacted, not found. As Latour and Woolgar proposed, “this bundle of out-thereness can be understood as an accomplishment rather than something that defines and sets limits to the ways in which we can properly know the world” (quoted in Law, 2004, p. 37). This is a more comprehensive and consequential view through which science produces the very things it studies. Facts, “bundles of out-thereness,” are the result of research, rather than discovered in research.

A simple, but striking illustration of enactment is a recent study of the effects of analytical variability. The “many analysts, one data set” project was a collaborative effort of 29 teams that each investigated the same research question (do soccer players with a dark skin tone get more red card bookings from referees than players with light skin tone?) with the same data set (Silberzahn et al., 2018). The teams used a variety of analytic strategies to answer this question, and despite two rounds of (online) discussion between the teams, there remained variability in the strategies. The differences made a difference: Although most teams found a relation between skin color and red cards, some did not; not all relationships were statistically significant, and the effect sizes varied. Depending on the analytical strategy one chooses, therefore, there is racism in soccer or there is not. The fact is coextensive with the analysis of the data.^2^ This is not to say that the fact is made up or that “anything goes.” Every analytic strategy has to find justification in the field of statistical methodology, a field known for its robust debates. Nor does it mean that race is not an issue in soccer. It clearly is: The fact that this particular research question is considered to be worth investigating is itself testimony to that. But turning this issue into a scientific fact is not so much a matter of discovering something already out there (racism in soccer) but of mobilizing particular statistical methods to detect patterns in the data, which are then sent out into the world of soccer as facts about racism. And these results can go on to set in motion changes in soccer: diversity initiatives, perhaps.

Alexandra Rutherford’s (2017) analysis of sexual assault surveys provides another example. These surveys are performative in that they “materialize experiences in new ways,” making certain experiences—and not others—“real” (2017, p. 115). Yet not any measure of sexual assault can materialize experiences, for such practices necessarily perform “within a complex assemblage of implicit and explicit beliefs, attitudes, institutions, communities, and politics (including, importantly, feminist politics)” (2017, p. 116). Neither does Rutherford mean that, as American conservatives like to claim, date rape is a fictitious phenomenon, created by feminist social scientists. Instead she describes how rape was given a particular *kind* of reality by the surveys that were central to the debate. The experiences of rape survivors became entangled with numbers collected to measure their prevalence. The surveys “materialized and rematerialized—via numbers and statistics—experiences that had been individual, private, unarticulated and—before the 1980s—unmeasured” (Rutherford, 2017, p. 114). It was in this quantified form—particularly the one-in-five statistic—that date rape became a topic in American culture, mediated by bureaucrats, policy-makers, and media.

An enactment perspective on science, then, holds to *realism* regarding the world and entities in the world while at the same time maintaining that scientific practices “bring (aspects of) the world into existence”: Certain realities are being enacted.^3^ From this follows a second feature of the enactment perspective. Given that entities are enacted in the course of scientific investigations, it is possible, even likely, that different practices can yield *different entities*. Entities and objects thus are never finished or complete things but rather are the effects of practices (Law, 2004; Law & Lien, 2013; Woolgar & Lezaun, 2013). Mol’s (2002) influential study of the medical diagnosis and treatment as well as patients’ lived experiences of atherosclerosis reveals that the numerous sites of diagnosis and treatment identify and engage different entities, not just different representations of or perspectives on the entity “atherosclerosis.” In these different sites atherosclerosis is different things. To connect them into different instances of a single entity requires work; it is not a straightforward “reflection of any innate commonality or characteristic” (Woolgar & Lezaun, 2013, p. 325). That work may be practical (connecting diagnostic results into a single dossier) as well as theoretical.

Third, given that the entities are generative effects of specific scientific practices, variations in scientific practices typically result in *differences*, *uncertainties*, and *precariousness* (Pickering, 2010). Scientific work involves ongoing efforts to reduce these uncertainties and multiplicities through techniques created and deployed to align data and to stabilize and produce or reproduce the entity (Guenther & Hess, 2016; Hoeppe, 2014). These efforts do not always succeed. For instance, analyzing the conceptions of “antisociality” and “psychopathy” and the proposed biomarkers for these disorders developed in British psychiatry between 1950 and 2010, Pickersgill (2014) found enduring uncertainties and diversity of both theories and methods; 60 years of investigations yielded neither scientific consensus nor even stable referents for the disorders. Contrary to conventional science’s epistemology, which would deem this a situation of problematic disunity, if not a crisis that warrants resolution, researchers and clinicians pursued these varied conceptions, undertaking “practical uncertainty work” but also remaining aware of the absence of consensus or clarity. Such a state of “ontological anarchy” actually proves to be generative, providing intellectual resources and degrees of freedom as well as entities for both researchers and clinicians to proceed with their practical endeavors. Pickersgill concluded, “Ontological anarchy is thus—to a degree—autopoietic: it is a response to the uncertainties inherent to dealing with antisociality in a psychiatric context, as well as an engine powering the generation of yet more ambiguity” (p. 147).

Fourth, although scientific entities are the effects of elaborate technical practices, that work can be transported and taken up outside investigative arenas and applied to social life, with the possible eventual effect of *changing human thought and behavior* (Hacking, 1995, 2007; MacIntyre, 1985; Richards, 2002; Stam, 2015). For example, through the uptake of the sciences and social sciences, the application of economic models to the financial world has performative effects “and among these effects is to alter economic processes to make them more like their depiction by economics” (MacKenzie et al., 2007, p. 67). Taking economics as the test case, MacKenzie (2007) identifies three variants of performativity. “Generic performativity” is the basic use of an economic idea. *Effective performativity* is the use of an economic idea that “makes a difference” in the world: Using the idea changes economic processes and realities. *Barnesian performativity* is a stronger version of effective performativity that results in the altering of actual economic processes to make them more like economics model or theory. By contrast, *counterperformativity* is use of an economic idea that changes economic processes so that they conform less well with the depiction provided by economics. The case of economics offers a tool for thinking about the ways that psychology travels and its entities are performed beyond scientific spaces. Evidence closely resembling Barnesian performativity was found by Haslam (2016) who tracked, both quantitatively and qualitatively, the circulation of psychological concepts in North American society. Haslam (2016) described the expansion of psychological concepts as “concept creep,” noting how they take shape and mutate in response not merely to scientific evidence but also psychologists’ political inclinations and changing social conditions.

Finally, the enactment perspective can be generalized to encompass not only scientific practice but also the world as a whole, thus turning this perspective into a metaphysics, albeit a different one than assumed by most scientists. The variability and multiplicity that are shown in studies of scientific practice are then taken to be characteristics of reality as such, whether science is involved or not. Instead of the usual Western metaphysical conception of reality as fundamentally singular, stable, and determinate, such an alternative metaphysics pictures reality as “an ultimately undecidable flux” (Law, 2004, p. 144).^4^ This is obviously a radical and potentially controversial idea, but it is directly relevant to the ideas of the proponents of conceptual replication. For them, flux is a fundamental character of the social world.

## Conceptual Replication as Enactment

A coterie of researchers has responded to the recent calls for more direct replication in psychology by contesting its efficacy and scientific value. Some have even challenged the very possibility of direct replications. Instead, they promote conceptual replications, which use different operationalizations, variables, experimental designs, and participants to test the theory of the original study. (It warrants note that although the two forms of replication—direct and conceptual—typically are considered distinct kinds according to “standard discourse” [Crandall & Sherman, 2016, p. 93], some authors describe fuzzy boundaries between them or give a more elaborate taxonomy of replication kinds.) Proponents of conceptualism forward a nuanced understanding of Popper’s work on scientific epistemology, especially regarding falsifiability and confirmation, and also cite other philosophers of science who report on the ambiguity and even logical impossibility of direct replication or emphasize how science is a collective, accumulating activity (Cesario, 2014; Crandall & Sherman, 2016; Stroebe & Strack, 2014). Supported by these philosophical positions, conceptualists argue that the primary function of replication is not falsification but exploration and development of theory, submitting that replication of a concept across different experimental situations is more robust than replications of exact situations. Advocates of conceptual replication generally prioritize basic over applied research, discovery over intervention, and exploration over confirmation. Beyond maintaining that direct replications (whether they disconfirm or confirm the original study) provide ambiguous evidence, they advance three fundamental and substantive claims: the context sensitivity of psychological phenomena and processes, the preeminent scientific goal of theory development, and the special expertise required of psychological scientists.

For conceptualists, failures of direct replication do not indicate that the hypothesis or theory is necessarily wrong but rather that many psychological phenomena and their experimental effects are highly sensitive to context and, therefore, often cannot be replicated exactly. They observe that psychology’s phenomena are affected by situation, culture, language, politics, and personal experiences. Thus, the effects observed via empirical investigations can vary over time and across situations. Such is the mutability and flux of (social psychological) phenomena that “one can never step in the same river twice” (Crandall & Sherman, 2016, p. 94). Extreme context-sensitivity is “the reality of our subject” (Dijksterhuis, 2014, p. 73). Conceptualists draw attention to two context-sensitive domains: the local context of the investigative situation and the larger one of cultural and worldly events. The identification of variations in investigative settings (typically experiments) echoes and amplifies a number of methodological concerns raised in discussions of reproducibility: participant populations and sampling, time, location, variations in instruments and stimuli, experimenter effects, and the like. Experimental manipulations in social psychology, for instance, might have “different psychological properties and effects if used in contexts or populations different from the original experiments”; exact replications, therefore, “can never be achieved” (Fabrigar & Wegener, 2016, p. 72) and “are fundamentally impossible in social-personality psychology” (Reis & Lee, 2016, p. 149). Experiments can never be repeated because “effect sizes are not determined in a universe that is purified of all other influences, observed strength is determined by both the systematic variance between and the error within the experimental conditions” (Strack, 2017, p. 2).

Variability stems not only from unavoidable micro-level differences across investigative situations but also from variations in culture, history, politics, and climate that can affect behavior, cognitions, and emotions. Conceptualists find this macro-level sensitivity to be unremarkable given that, per Bavel (2016b, p. 4936), “the notion that human psychology is shaped by the social context has been the central premise of the field (social psychology) for nearly a century.” Likewise, confirming cultural sensitivity is our knowledge about the evolved complexity of the human mind (Cesario, 2014). Taking seriously the cultural, environmental, and historical influences on psychological processes presents implications that extend beyond reproducibility of science to core questions about the very nature of psychological phenomena: They are matters of ontology. Many phenomena are moderated by cultural and historical conditions and sometimes might even be “culture dependent” (Stroebe et al., 2012, p. 679). To Iso-Ahola (2017), “all of this means that there are no static phenomenon particles, unlike the Higgs Boson particle in physics” (p. 2).

Some have suggested that such heightened concern about the nature of psychological phenomena is more serious than earlier crises in social psychology. Whereas researchers once questioned whether a phenomenon existed outside the lab, “the question being asked today is much more unsettling: ‘does this phenomenon exist at all’?” (Hales, 2016, p. 40). However, most conceptualists maintain that there are limits to the flux or changeability of psychological phenomena. They hold either that not all phenomena are dependent on cultural factors or that “the brain, behavior, and society are orderly in their complexity rather than lawful in their simplicity” (Bavel et al., 2016a, p. 6458), or that despite variability, essential psychological phenomena can be located through theory-guided research. Behaviors and thoughts are the effects of numerous, sometimes imperceptible, unseen and mediating factors—“underlying” or “internal” mechanisms. The path toward discovering these essential mechanisms is not via refinement of direct-replication techniques, although their improvement is important, but through intensified theory development. “Confidence in theory” is valued over the “confidence in operationalizations” of researchers conducting direct replications (Crandall & Sherman, 2016, p. 93).

Prioritizing theory over a concerted project to replicate experiments and reproduce effects is warranted by epistemological claims. Most basic of these claims is that reproducibility of an empirical finding is less valuable than evidence of validity of a theory (Greenfield, 2017; S. B. Klein, 2014; Stroebe & Strack, 2014). (Given this spotlighting of theory, Zwaan et al. [2018] suggest that the term “conceptual” is a misnomer and that a more appropriate designation would be “extension” to refer to testing and extending theory.) Advocates of conceptual replication stress the cumulative nature of science not as amassing countless empirical findings but as progressing through theory development, refinement, and sometimes replacement. Crandall and Sherman ask fellow researchers to “trade higher confidence in a single set of operations for higher confidence in theory” (2016, p. 98). They note that not data but “ideas are the unit of analysis in conceptual replication” (2016, p. 95). Science is understood as a collective enterprise composed of research programs for which the goal is creating valid theories (Reis & Lee, 2016; Stroebe, 2016).

According to Strack and Stroebe (2018), the goal is to understand underlying mechanisms, which requires not only experimenting but also working at “the theoretical level” (para. 5). So relying on theory entails appreciation that theories “are formulated on a level that transcends the concrete evidence; and their validity does not rest on the outcome of one specific experimental paradigm” (p. 39). In contrast to reformers, conceptualists engage the psychological world not at the ground level of objects, behaviors, or effects. Hacking (1999) finds use of what Willard Van Orman Quine called “semantic ascent” (as quoted by Hacking, 1999, p. 21), shifting attention from ground-level talk about objects to the abstract level of talk about what those objects mean. To Hacking, such ascent entails the use of “elevator words” (p. 21): The conceptualists foreground theory (over data), special expertise (over routine science training), and concepts (over behaviors and effects). Without making this ascent, conceptualists intimate, staying at ground-level absorption with direct replications risks ambiguous outcomes and perhaps more importantly produces effects specific to the experiment’s unique conditions. However, along with this ascent to theory and expertise is an expectation of ultimately locating basic psychological mechanisms, presumably grounded in neurological processes.

Along with promoting a theory-driven enterprise on epistemic grounds, conceptualists also champion theory on ontological grounds. An observed phenomenon or effect is not necessarily evidence of the “underlying mechanisms” (Stroebe & Strack, 2014, p. 59). The “collection of effects and phenomena” deters researchers from exploring basic laws (Strack, 2017, p. 3). In other words, the cultural and contextual sensitivity of psychological processes and the unseen moderators pose formidable challenges to ambitions regarding direct replication. Conceptual replications instead aim to “operationalize the underlying theoretical variables using different manipulations and/or different measures” (Stroebe & Strack, 2014, p. 60). It is precisely through variations on an earlier study (i.e., through conceptual replications) that the underlying stable reality can be brought into view. Foolishly replicating the same procedure (a direct replication) only risks failure: An experiment that once produced an effect may never do so again because of the ever-changing context (Crandall & Sherman, 2016). Social psychologists are condemned to continuous variation if they want to keep a hold on the stable psychological reality.

Another contention is that researchers conducting direct replications underappreciate scientific expertise. Whereas the projects initiated to foster direct replication assume that well-trained researchers can proficiently conduct replications, the conceptualists often mention the necessity of special “expertise and diligence to generate a new result in a reliable fashion” (Strack, 2017, p. 3; Bavel et al., 2016a). Some are remarkably critical, suggesting that “the replication crisis can even be seen as rewarding incompetence” through reformers’ supposition that any researcher can undertake replications; in contrast, Baumeister avers, competence requires “years of specialized training and skill cultivation” (2016, p. 156). Replications depend on expertise in the specific subject area, and without this extensive experiential proficiency, “replication experts” “may train a big telescope with a dirty lens on the wrong planet” (Schwarz & Clore, 2016, p. 1409). Indeed, sometimes researchers have responded to failed replications of their original studies with comments on the replication researcher’s lack of necessary expertise (e.g., Schnall, 2014). Juxtaposed against reformers’ unease about researchers’ “degrees of freedom” or nonstandard research decisions, is conceptualists’ valuing of researchers’ expert judgment. Using terms from Daston and Galison’s (2007) history of objectivity, the conceptualists emphasize “trained judgment” (the crucial value of special expertise) over the faith in “mechanical objectivity” (rigorous, routine procedures) of researchers conducting direct replications.

There are two ways that one might appreciate the conceptualist position via enactment. From one angle, conceptualists practically mirror enactment theory with their strong emphasis on chance, variability, and flux in social behavior. Such an enactment perspective is illustrated in the recognition that “even a technically identical manipulation does not guarantee an equivalent test of psychology phenomenon when the context changes” (Schwarz & Clore, 2016, p. 1408). However, this is not the reality of significance to conceptualists, and their enactments transpire in a different place and quite differently—as internal or underlying mechanisms. An underlying psychological reality, dynamic yet lawful, consists of sometimes imperceptible, unseen factors. Conceptualists hold to a belief in the lawfulness and stability of reality, a reality that can be known through crafting good theory: “Empirical outcomes are meaningful only with respect to the theory being tested” (Stroebe & Strack, 2014, p. 60). Theory building is a crucial form of the work required to make entities singular. A theory connects different effects and different results from studies run by various researchers working at various sites and at different times, and makes them evidence of a single mechanism, process, or disposition.

An example of the work of singularization is Barsalou’s (2016) assessment of the varying forms and findings of social priming, including evidence of individual differences. He found that “simple direct pathways from primes to primed responses rarely, if ever, exist” (p. 9). Given such cognitive and behavioral complexity, he proposes a theory of “situated conceptualization” that provides a “natural” and “principled account of the knowledge structures that develop” in the form of individuals’ multimodal inferences (p. 9). The theory holds that the brain processes different elements of a situation (e.g., place, agents, objects, self, action) and multiple experiences and, over time, integrates these experiences and produces conceptual interpretations. These “situated conceptualizations” are activated later when the individual encounters a situation containing elements of earlier ones; there ensue pattern completion inferences that are implemented through multimodal (of place, agents, objects, etc.) simulations in the brain. This multistage theory explains the inadequacy of direct replications in the study of social priming: “Because any aspect of these situated conceptualizations can trigger this process, or be the outcome of it, social priming takes infinitely many forms” (p. 8). Yet the theoretical framework gives an account of the fundamental processes that can yield highly variable effects.

Another project aiming toward singularization, but of a different kind, is Greenfield’s (2017) proposal to move beyond the issue of whether a phenomenon is replicable to study the effects of sociodynamic changes on culture and behavior. Her “theory of social change and development” (p. 763) offers a description of changes on several levels, from sociodemographic down to behavioral, and the causal influences going from the higher to the lower levels. Greenfield discusses two failed replications of social psychological experiments and argues that her theory explains both failures. They were not failures to replicate but rather demonstrations “of the effect of culture change on behavior” (p. 768).

The theory work being promoted is not abstract theorizing; it works to bring associated enacted entities together to be understood as the same thing. According to Woolgar and Lezaun (2013) and others, the production of singularity is always a fragile achievement and often a source of tension. In fact, some conceptualists admit that researchers cannot always attain consensus about the meaning of their conceptualizations. To prevent such problems, Crandall and Sherman advise “careful pilot testing” and “robust manipulation checks” (2016, p. 98); Strack and Stroebe (2018) advise using “theoretically grounded hypotheses that generate specific predictions” (para. 7). The conceptualists’ attempts to attain consensus or realize singularity regarding the entities being investigated remains fragile, however, because direct replications, following enhanced methodological guidelines, keep yielding findings that differ from the original results.

## Direct Replication as Enactment

Ever since the reform movement started to gather steam in 2011, replication has been a central concern. There are several aspects related to the role of (direct) replication in science according to the reformers. First, the reproducibility of events is often presented, following Popper, as a precondition of falsifiability and thus of science. Conceptual replication is important (for the refinement and further development of a theory), but only after the reproducibility of the effect under the same investigative conditions has been determined. “If a phenomenon is not replicable (i.e., it cannot be consistently observed), it is simply not possible to empirically pursue the other goals of science” (LeBel et al., 2017, pp. 8–9; Earp & Trafimow, 2015). Second, replication must be possible by following explicit instructions, given sufficient expertise—by “anyone who has learned the relevant technique,” as Popper (2002, p. 81) put it. Reformers are very skeptical about appeals to the need for more than standard technical skills (Neuroskeptic, 2014; Srivastava, 2014b; Wilson, 2014). Third, (direct) replication is seen as providing evidence for the reality of a phenomenon. Reproducibility shows the robustness and reliability of a phenomenon.

The reformers’ efforts to create a scientific practice based on direct replication are characterized by attention to statistical and methodological detail; by an emphasis on rules, regulations, and administration; and by the important role of infrastructure. The statistical and methodological inadequacies and errors of the current practice have been listed in impressive detail (Forstmeier et al., 2017; Wicherts et al., 2016) and are generally seen to consist in researchers’ exploitation of so-called researcher degrees of freedom (Simmons et al., 2011) to arrive at the desired result. In every scientific study, many decisions have to be made (e.g., regarding sample size, which comparisons to test, which tests to report). Such decisions can have a great influence on the study’s results, as shown, for example, by Simmons et al. (2011), and opportunistic use of this flexibility increases the chance of false positives. The solution that is most commonly proposed is to constrain this freedom by directing the researcher to make these choices before data collection and publicly register the study design and data analysis plan that has an electronic date stamp as validation. This is called preregistration (Wagenmakers et al., 2012).^5^ In the related Registered Report (RR) format, a journal editor guarantees publication of a study if the preregistered study plan is reviewed positively, regardless of the eventual results of the study (Chambers, 2013). Thus, in an RR, both researchers and editors constrain their freedom in the interest of falsifiability, giving space to negative results and their publication.

Preregistration is an administrative procedure for reducing or making transparent the liberties researchers might take in data analysis. An administrative gesture with a similar purpose was proposed by Simmons et al. (2012). Their “21 word solution” requires authors to state that “We report how we determined our sample size, all data exclusions (if any), all manipulations, and all measures in the study” (2012, p. 4). Another kind of “statement” was proposed by Simons et al. (2017). The “constraints on generality” statement would have researchers declare to which population they claim their findings can be generalized, so that researchers conducting replication studies can take this into account. Finally, a related, declaration-type of gesture are the open science badges and the associated standards introduced by OSF (Blohowiak et al., 2013). Whereas the 21-word solution and the constraints-on-generality statement have not (yet) found wide use, the badges are implemented by an increasing number of journals and have been claimed to be effective in encouraging preregistration, data sharing, and other open practices (although this claim has been contested by Bastian, 2017).

Online infrastructure is an important part of this research practice. OSF (https://osf.io) facilitates a transparent, collaborative research process from inception to publication, including preregistration of study plan. There are online inventories of replication results (http://curatescience.org and the older http://www.psychfiledrawer.org). There is a preprint archive for psychology (https://psyarxiv.com, modeled on https://arxiv.org) allowing the quick dissemination of manuscripts and their discussion by the community. Social media—Twitter and Facebook, in particular—is a forum where developments are discussed almost instantaneously, by the widely dispersed community, without much hierarchy or gatekeeping. Finally, many reformers have blogs, where they formulate opinions, present results, comment on others’ work, continue discussions that started on Twitter, or start debates that spill over onto Twitter.

Together, the statistical and methodological rules and strictures, the registration and archiving of decisions and designs, the statements, the badges with their standards, the repositories of results and manuscripts, the online collaborative spaces, and the social-media communication infrastructure, form a large, heterogeneous device—a “method assemblage” as Law (2004) calls it—for the production of “reproducible science” (Munafò et al., 2017, p. 1). The operation of this device has resulted in serious doubt being cast upon accepted theories and effects in social psychology; several social priming effects (Doyen et al., 2012; Shanks et al., 2013) and power posing (Ranehill et al., 2015), among others, have been thrown into doubt by failed direct replications. Proponents have lauded this corrective role of direct replications and see it as falsification in action, whereas others have been critical of failed replications of their work (Bargh, 2012; Schnall, 2014) and/or have condemned what they see as the negative and hostile attitude of some reformers (Baumeister, 2016; Fiske, 2016; Hamlin, 2017).

There has been much debate over whether direct replication and its proponents play a corrective or a destructive role. However, in our view, the science of the reform movement is better seen as performative and productive of reality—as enacting a reality. Reformers take the production of phenomena very seriously. An example of the performativity of their approach to research is the replication by Wagenmakers et al. (2016) of Strack et al.’s (1988) facial-feedback experiment. The facial-feedback hypothesis states that the facial expression of an emotion will intensify or even bring about the experience of that emotion itself. Strack et al. tested the more specific hypothesis that this effect occurs even without cognitive mediation (i.e., when people are not aware they are expressing a certain emotion). To this end, they devised a bogus experimental task that made participants unwittingly create an expression (a smile or a pout). Specifically, participants were asked to rate the funniness of cartoons on a paper questionnaire with a pen that they held either between their teeth (smile), between their lips (pout), or in their nondominant hands (neutral) as part of what they were told was “an experiment investigating people’s ability to perform different tasks with parts of their body not normally used for those tasks, as injured or handicapped persons often have to do” (Strack et al., 1988, p. 770). In the smile condition, cartoons were rated funnier than in the other two conditions.^6^

Note that Strack et al.’s experiment is itself a study of enactment, revolving as it does around the question of whether enacting an emotion by expressing it creates the reality of that emotion. Correspondingly, the report dwells extensively on how to direct the performance of the subjects. The description of the experimental procedure is lengthy, including the precise wording of the cover story, the instructions that the subjects received about how to hold the pen (illustrated with two photographs), what type of pen was used, and the fact that the four Gary Larson cartoons that were used had been “prerated as being moderately funny” (Strack et al., 1988, p. 771). There was also a pretesting procedure to make sure that the instructions produced the kind of spontaneous performance that was intended: one in which participants were not aware of the purpose of the experiment.

These performative aspects of the facial-feedback experiment, already prominent in the original study, are further emphasized in the replication. First of all, great care was taken so that the script of the experiment reproduced the proper performance of the participants. Strack provided the original experimental materials and gave feedback but declined to review the protocol. Ultimately it was vetted by another researcher with experience with this experimental task, and it was then preregistered on OSF. Second, because this replication study consisted of 17 separate replication experiments in different labs, the coordinators of the collaboration made sure that the participating labs received identical, detailed instructions accompanied by a video of “the complete 24-step procedure” (Wagenmakers et al., 2016, p. 919; video available at https://osf.io/spf95/). Care was taken that translations of the research materials were accurate by having “a separate bilingual speaker independently translate them back to the original language” (Wagenmakers et al., 2016, p. 919). Third, the replication study included several enhancements of the original experiment intended to improve the participants’ performance. The participants received part of their instruction by video (to prevent experimenter-expectancy effects), and they were filmed while they were doing the experimental task to check that they held the pen correctly. Moreover, the researchers took care to select participants who were unlikely to be familiar with the original study, so that their performance would be spontaneous.

This meticulously staged, precisely choreographed, 17-experiment study produced no statistically discernible difference between the smile condition and the pout condition. The 17 effect sizes (mean rating differences between the two conditions) were small, having a meta-analytic effect size of .03 (Wagenmakers et al., 2016). It would be a mistake, however, to think that “nothing” came out of this study. Not only is a null result still a result (as every statistician would emphasize), but in terms of performativity, something real was enacted here, meticulously and abundantly. The 1,894 participants all took a pen in their mouths in either of two very specific ways, looked at a set of Gary Larson cartoons, and indicated their level of amusement on a piece of paper with that pen. On average, these participants were moderately amused, whichever way they held the pen in their mouths. Superficially this reality is nothing new: The manipulation did not affect amusement, reality was not transformed. But it was a performance that was both richer than the original in terms of number of actors and their geographical spread, as well as more homogeneous: Regardless of condition, everyone acted the same on average. It may not be very interesting at face value, but it is powerful in its overwhelming uniformity.

Yet the uniformity was not perfect. Although Wagenmakers and colleagues had connected the 17 replication efforts into one singular null result, Strack pointed out cracks in the uniform facade. He argued that the studies that had employed nonpsychology students as participants collectively did have a significant effect, in the expected direction, possibly because these students were unaware of the existence of the facial-feedback effect (Strack, 2016). In general, it seemed significant to him that nine teams found an effect in one direction and eight teams an effect in the opposite direction (Strack, 2017). He also pointed out that filming the participants during the replication experiments might have made a difference: The camera could have made the participants self-conscious about their performance, inhibiting their amusement (Strack, 2016). This hypothesis has subsequently been tested by Noah et al. (2018), who found that the presence of a camera indeed eliminated the facial-feedback effect. Wagenmakers & Gronau (2018) and Gelman (2018), however, expressed reservations about Noah et al.’s replication study.

To get more clarity, a meta-analysis of 138 facial-feedback studies was conducted that examined the overall effect of facial feedback and the influence of 12 moderating variables. There were effects of facial feedback on emotional experience, but they tended to be small and highly variable, for reasons that the meta-analysis could not elucidate (Coles, Larsen, & Lench, 2019). Contrary to what Noah et al. (2018) found, video recording the participants hardly made a difference. Another multilab replication project is now under way to shed more light on facial feedback and determine when it should have a reliable effect on emotion (Coles, March, et al., 2019). The performance of the participants gets even more attention than in the original study and the replication by Wagenmakers et al. (2016): Participants produce facial expressions in three different ways (including mimicking the expressions of actors “displaying prototypical expressions of happiness”; Coles, March, et al., 2019, p. 7), and rating their own performance in four different ways. In three pilot studies, facial-feedback effects could be reliably produced, but not with the pen-in-mouth task. It is not clear why. Thus, the multilab replication effort by Wagenmakers et al. of the pen-in-mouth study set in motion further discussion and research that have produced a view of the connection between facial feedback and emotion that is considerably messier than was the case before 2016. Whereas Strack saw one general facial-feedback hypothesis confirmed by many different studies, the current state of the field is one of multiple effects that vary in strength for reasons that are largely unknown, loosely connected by the fact that they show that facial feedback generally seems to have a small effect on emotion.

## Enacting Variability

Other multilab replication efforts have had a similar effect of creating “mess.” Many Labs 2, for example, conducted replications of 28 original findings, using 125 samples with a total of 15,305 participants in 36 countries (R. A. Klein et al., 2018). Only 15 findings could be replicated. Contrary to the conceptualists’ common explanation that nonreplications may be due to variability in the cultural context of the participants or the expertise of the researchers, for the most part, effects could either be reproduced or not; lab or sample hardly mattered. There was, however, some heterogeneity in the effect sizes, particularly among the effects that were larger on average. Thus there was still some variability, but not where conceptualists would expect it, in differences between labs or cultural contexts. The conceptualists’ argument, that “manipulations and measures often derive their meaning from the historical, social, and cultural context at a given time” (Stroebe, 2019, p. 95) and a failure to reproduce an effect in a direct replication is therefore uninformative, is problematic in light of these results.

Olsson-Collentine et al. (2020) determined the heterogeneity in the sizes of 68 effects produced in preregistered, multilab, direct-replication studies and found it to be small or zero in most cases. In other words, if you maximize the similarity in procedure, remove researcher degrees of freedom, but conduct the study in different labs (or online), in different places and countries, with different samples, effect sizes tend to be quite similar. But Olsson-Collentine et al. also note that for 12 out of 68 effects, heterogeneity was large, particularly for large effects. Moreover, variability is restricted here to sample and settings, but most of the samples were undergraduates, and the (immediate) settings were university labs. There are other potential sources of variability. Commenting on Many Labs 2, Srivastava (2018) has argued that that study did not prove that social behavior is not contextually (historically, culturally) variable. It shows that there usually are no *hidden* moderators lurking in experiments. Psychologists’ efforts at experimental control are usually successful. That means, Srivastava concluded, that if you believe in contextual variability, you have to purposely study it, rather than merely draw on it as a possible explanation of replication failures. Forscher has similarly stressed that social psychologists need to do more than their usual “small-ish one-shot experiments using pallid manipulations of dubious validity” (2018b) to produce situational influences on behavior. Instead that may require going out of the lab to “leverage naturally occurring experiments” (2018a) and doing longitudinal studies.

Congruent points of view have been put forward earlier in response to fundamental problems in the discipline. Consider for example Greenwald et al.’s (1986) article “Under What Conditions Does Theory Obstruct Research Progress?” Their diagnosis of the state of the discipline in the mid-1980s resembles that put forward by current reformers. They noted that the academic incentive structure and the publication practices that psychologists have to work with encourage a strong confirmation bias in their research practices, which in turn leads to methodological problems. “Researchers’ dispositions to confirm hypotheses support their use of methods that are demonstrably prone to misinterpretation and, because of that, obstruct scientific progress” (1986, p. 222). Their solution to these problems was to shift the aim of research from theory to results. Psychological research should be “condition-seeking”: Rather than testing theory (or, in practice, seeking its confirmation), it should look for the conditions under which a psychological phenomenon occurs (1986, p. 223). In such an approach, theory is an instrument rather than a goal in itself. It gives direction to the condition-seeking process and keeps it from devolving into the simple, unstructured accumulation of qualifications of the general theory.

Greenwald et al. (1986) were inspired by McGuire’s contextualism (later renamed *perspectivism*), according to which every conceivable hypothesis in psychology is true in some context, and the research process consists of discovering that context and describing it in detail. Sharing a contextualist premise that knowledge emerges in contexts that are dynamic, McGuire’s perspectivism then reasons that “all hypotheses are true in the sense that a reasonably ingenious and persistent scientist with sufficient resources can always finally create or find some special context in which the hypothesized relationship obtains” (McGuire, 1986, p. 284). Thus, any empirical claim “has potential for simulating its referent adequately in some contexts and from some perspectives” and “any hypothesis adequately represents the known from some viewpoints but not from others” (p. 281). His epistemic guide for expanding and clarifying hypotheses understands research as a “creative performance” (p. 293) that exploits rather than constrains the “revelatory power” (p. 297) of both empirical and theoretical work. Empirical research does not test the truth of a theory but aims to develop the theory by exploring the conditions in which a phenomenon occurs. Greenwald et al. (1986) distinguish their proposal from McGuire’s by saying they go beyond his ideas “primarily in concluding that theory testing should often be displaced from its status as a central goal of research” (p. 226).

A similar emphasis on phenomena and their context can be found in Paul Rozin’s critique of social psychology. Following Solomon Asch, Rozin contended that social psychology’s attempt to emulate the rigor and precision of the natural sciences has remained fruitless because it was not preceded by “an extensive examination and collection of relevant phenomena and the description of universal or contingent invariances” (Rozin, 2001, p. 3). It is useless to test a hypothesis, however rigorously, if it is not informed by a thorough exploration of the phenomena of interest.^7^ Social psychology tries to ascend toward theoretical abstraction and formalization without a solid grounding in real-world phenomena.^8^ Instead, experiments in social psychology are usually oblivious to context, seemingly transcending “time, location, culture, race, religion, and social class” (2001, p. 4). Their results are often difficult to generalize and have no obvious bearing on practical, everyday problems.

An elaborate call for contextualism was forwarded in the edited volume *Contextualism and Understanding in the Behavioral Sciences* (Rosnow & Georgoudi, 1986a). The editors ground contextualism with the premise that social reality is active and ongoing; therefore, “all knowledge is perennially conceptual and conjectural and no method can conclusively demonstrate the ‘truth’” (Rosnow & Georgoudi, 1986b, p. 4). That psychology’s facts are indeterminate, however, does not preclude their empirical scrutiny. Further and importantly, in this contextualist perspective, context is not an “independent ontological entity” for context and act are integral to each other. And the editors take methodological pluralism as necessary to investigate “the wider context that ‘allows’ or ‘invites’ the occurrence of that event and renders it socially intelligible” (p. 5). Scientific method does not stand outside this contextual web to detect entities but is itself an active and productive process. Thus, “Both the products of this process, as well as the process itself, will reflect the contextual boundaries in which they operate or develop” (p. 18).

The enactment perspective goes beyond these proposals in its rejection of the discovery metaphor, instead seeing research as productive of reality. In our opinion, the current crisis discussion is pointing in precisely this direction, despite the generally rather traditional philosophical assumptions of both conceptualists and reformers. The emphasis of the proponents of conceptual replication on the variability of human behavior and on the multiple constituents of psychological phenomena is not incompatible with the attention to procedural detail of the advocates of direct replication. Our proposal is not merely to do away with the dichotomy of direct versus conceptual replication. We agree with Nosek and Errington (2020) that this distinction is unhelpful. Because no two studies can be identical, no replication “exact,” the claim that one study’s methods replicate another study’s methods requires criteria for the relevance of differences (Nosek and Errington, 2020). A study replicates another study in some sense, and that sense is supplied by theory.^9^ We believe that social psychology requires a broad spectrum of replication studies, and that spectrum cannot be neatly divided into “direct” versus “conceptual.” Most of all, however, we think social psychology needs to be geared to producing multiple psychological realities rather than discovering a single psychological truth. It is a shift from discovery to technology, from “mirroring to world-making” (Gergen, 2015, p. 287). It is a shift away from the seemingly endless proliferation of “functional entities” that researchers produce and eventually discard as new ones are introduced (Stam, 2010). Such a scientific program would combine an interest in variability with a focus on concrete effects and the minutiae of their production. As the previous few years have made abundantly clear, it is precisely through paying close attention to whether, when, and how effects are replicated that the reality performed in social psychology becomes fragile, variable, and messy. That in turn invites the consideration of other approaches to research, beyond the traditional laboratory experiment, and beyond the search for basic principles of social behavior.

Such a shift suggests the need to reflect on the political as well as the pragmatic aspects of psychological research and the realities it produces. It puts to question the binary of “basic” and “applied” research that is generally presumed by both reformers and conceptualists. If research is no longer conceived of as the discovery of an objective reality but rather as the generation of diverse realities, then what realities we choose to bring into being is a political and ethical as well as a scientific matter (Law, 2004; MacIntyre, 1985; Stam, 2010). In the case of the facial-feedback controversy, for example, it is remarkable that the practical relevance of the effect, if there is any, is largely ignored in the discussion.^10^ In general, we need to pay more attention to the reality we are making as we are doing our research, talking about it in TED talks, writing about it in the newspaper, and using it in our profession, and pay less attention to the search for a theory that will represent reality.

## Conclusion

The crisis literature is densely populated with charges of bad science, reports of one or another methodological deficiency, and multiple, technologically instituted directives for realizing robust psychological science, which, in turn, ultimately yield stronger truth claims. The various debates have produced a bifurcation of perspectives and the emergence of two prominent camps. One notably vocal group advocates direct replication (along with a host of other regulatory measures) as means to discover phenomena the existence of which is confirmed through their reproducibility. The other group advocates what can be understood as plural methods as a necessary means to discover psychological phenomena that they take to be dynamic and highly dependent on context. The focused, ongoing attention to what constitutes proper methods has often overshadowed the different ways in which these two camps think about ontology—about the nature of psychological entities. We suspect that the trenchant methodological disputes and differences in underlying ontological commitments will not be resolved solely through empirical work. Instead, a generative and reparative approach is to understand how research enacts realities that are generated through rigorous thinking, technical operations, instruments, trained judgment, and tact. Experiments perform certain realities that can be supported or challenged in subsequent empirical work. So understanding the enactment of psychological realities underscores the importance of plural methods and invites reconciliation by providing a set of questions (what reality is performed here, to what end, etc.) on which both camps can focus and that can constructively move them beyond the direct versus conceptual discussion.

That opposition of direct and conceptual replication, and the way they are commonly associated with emphases on permanence and variability, respectively, is unhelpful. Direct replication is necessary not only to detect flexibility in methods but also to demonstrate variability. To determine whether a phenomenon is context sensitive, one must try to produce it using the same procedure in different contexts. If it does vary, one can proceed to study this variability with studies that intentionally change this or that aspect of the original study.^11^ There is no inherent contradiction between rigorous, precise replication that seeks to control for flexibility and an interest in the variability of social behavior. We do think that that variability calls for methodological pluralism and, above all, for an awareness of the performativity of psychological research. Rather than persevering in a quest for stable mechanisms underlying the variability, it is better to embrace the variable phenomena psychology produces and take responsibility for them. With this enactment perspective and the consequent understanding of the roles of direct and conceptual replication, psychology’s future would be more phenomenon-centered and better able to determine under what conditions phenomena are enacted.

This has implications for the politics and ethics of psychology. These implications complicate even as they expand upon Miller’s (1969) long-revered call for “giving psychology away” (p. 1071) to improve “human welfare.” Psychology’s part in the making of the world, its ethical and political effects, has been long noticed if rarely acted upon. Reflecting on the ways that psychology makes its objects true (or false), MacIntyre (1985) called for psychologists’ attention to how “psychology has changed the human world in the course of interpreting it and created new phenomena in the course of trying to understand old ones” (p. 902). Psychology’s effect on culture “has been to foster types of character and modes of action,” an enormous effect that MacIntyre suggests raises and extends psychologists’ responsibilities. The ethics attending the realities that psychologists produce were recently examined in Stam’s (2015) call for an “ethics of shared understandings” (p. 117) and Haslam’s (2016) study of “concept creep” (p. 1). As Haslam concludes his analysis, understanding the drivers of concept creep “and evaluating its costs and benefits are important goals for people who care about psychology’s place in our cultures. Equally important is the task of deciding whether the trend should be encouraged, ignored, or resisted” (p. 15).

The stakes of electing one ontological perspective over the other (or others) and thus privileging one method over others are high. Alternatively, appreciating psychological research as enacting realities, and appreciating different methods as potentially producing different realities makes way for a genuinely open science, generative research programs, expanded reflection on ethics, and ultimately more richly informed, constructive scientific exchanges about the nature of psychological entities.

## Transparency

*Action Editor:* Adam Cohen

*Editor:* Laura A. King

## Notes

1. Nosek and Errington (2020) have argued that many “conceptual” replications are not in fact replications, but generalizations. They define a replication as a study the outcome of which is diagnostic with respect to an earlier study, both when the results confirm the claims of that earlier study and when they disconfirm them. According to Nosek and Errington (2020), however, conceptual replications are usually “not designed such that a failure to replicate would revise confidence in the original claim” (p. 7), and they are therefore not replications at all. Crandall and Sherman (2016) are strong proponents of conceptual replication but do think that failed conceptual replications should receive more attention than they currently do.

2. To which one could add that the “many analysts” project itself was also performative because it enacted the variability of analytical strategies and outcomes in a particular way. A different procedure in the project, or a different research question or data set, might all have resulted in different kinds and levels of variation between the teams.

3. These realities are not constructed, for there is an important difference between the notion of *social construction* and that of *enactment*: “the former describes social processes that result in durable realities, while the latter describes practices in the here and now that produce ephemeral effects—effects essentially coextensive with the practices that create them” (Woolgar & Lezaun, 2015, p. 463).

4. For a similar metaphysics, see Barad (2003).

5. Another solution is a so-called multiverse analysis, in which all raw data are processed in all possible, reasonable ways, and the resulting set of data sets is statistically analyzed (Steegen et al., 2016).

6. The study consisted of two experiments; the second tested several additional hypotheses. Strack et al.’s Experiment 2 is not discussed here because Wagenmakers et al. did not attempt to replicate it.

7. The same point was made by Eronen and Bringmann (2021): “In psychological science, there is not enough knowledge of robust phenomena to impose sufficient constraints” (p. 780) on theory development.

8. Van Rooij and Baggio (2021) contend that the real world capacities that psychology is about are basically known already, but it remains to explain them. We agree with the focus on the real world, but not that all “capacities” are known.

9. Since the theory is at the same time being tested, this leads to the “experimenter’s regress,” formulated by Collins (1985): The theory is both under investigation and is a criterion for a proper investigation.

10. Strack, however, has mentioned that research into facial feedback has led to the development of a treatment for depression with Botox to suppress frowning. This seems to us an application of this research that is important to discuss in terms of performativity. Coles and Larsen (2021), moreover, contest the quality of the evidence for the efficacy of Botox in treating depression.

11. See also Nosek et al. (2021), who write that “replications foster unplanned discovery of potential invalidity when an apparent replication produces a different result and stimulates theorizing about why the original and replication studies differed” (p. 7).
